# Oral HPV16 Prevalence in Oral Potentially Malignant Disorders and Oral Cavity Cancers

**DOI:** 10.3390/biom10020223

**Published:** 2020-02-03

**Authors:** Kai Dun Tang, Lilian Menezes, Kurt Baeten, Laurence J. Walsh, Bernard C. S. Whitfield, Martin D. Batstone, Liz Kenny, Ian H. Frazer, Gert C. Scheper, Chamindie Punyadeera

**Affiliations:** 1 Saliva & Liquid Biopsy Translational Research Team, The School of Biomedical Sciences, Institute of Health and Biomedical Innovation, Queensland University of Technology and the Translational Research Institute, Queensland, Brisbane, QLD 4059, Australia; kai.tang@qut.edu.au (K.D.T.); applegate2k@yahoo.com (L.M.); 2 Janssen Diagnostics, a division of Janssen Pharmaceutical NV, 2340 Beerse, Belgium; KBAETEN1@its.jnj.com; 3 School of Dentistry, The University of Queensland, Herston, QLD 4006, Australia; l.walsh@uq.edu.au; 4 Logan Hospital Integrated Specialist ENT Service, Metro South Health Service District, Queensland Health, Meadowbrook, QLD 4131, Australia; Bernard.Whitfield@health.qld.gov.au; 5 Department of Maxillo-Facial Surgery, Royal Brisbane and Women’s Hospital, The University of Queensland, Butterfield St, Herston, QLD 4029, Australia; Martin.Batstone@health.qld.gov.au; 6 Royal Brisbane and Women’s Hospital, Central Integrated Regional Cancer Service, The University of Queensland School of Medicine, Queenslands Health, Brisbane, QLD 4029, Australia; lizkenny@bigpond.net.au; 7 Faculty of Medicine, The University of Queensland, Translational Research Institute, Brisbane, QLD 4102, Australia; i.frazer@uq.edu.au; 8 Janssen Vaccines & Prevention BV, 2333CN Leiden, The Netherlands; GSCHEPER@its.jnj.com

**Keywords:** human papillomavirus, oral potentially malignant disorder, oral cavity cancer, saliva, viral load and HPV integration

## Abstract

The role of human papillomavirus type 16 (HPV16) in oral potentially malignant disorders (OPMD) and oral cavity carcinoma (OC) is still under debate. We investigated HPV16 prevalence in unstimulated saliva, oral rinse samples, oral swabs and tumour biopsies collected from OPMD (*n* = 83) and OC (*n* = 106) patients. HPV16 genotype, viral load, physical status (episomal vs. integrated) and tumour p16INK4a expression were determined. Oral HPV16 prevalence was higher in OC than in OPMD, but this difference was not statistically significant (7.5% (8/106) versus 3.6% (3/83), odds ratio (OR): 2.18, 95% confidence interval (CI): 0.56, 8.48, *p* = 0.26). There was a significant association (*p* < 0.05) between oral HPV16 infection and heavy tobacco consumption. Real-time PCR results indicated that no integration events occurred in either OPMD or OC cases based on the HPV16 E2/E6 ratio. HPV16 positive OPMD and OC patients had similar HPV16 E2 and E6 viral loads. The inter-rater agreement between tumour p16INK4a expression and oral HPV16 infection was considered as fair (k = 0.361) for OC. Our data suggest that the involvement of HPV16 in oral carcinogenesis is limited.

## 1. Introduction

Oral cavity carcinoma (OC), which includes carcinomas of the mucosal part of the lip, is the most common subset of head and neck carcinomas (HNC), affecting approximately 300,000 patients annually worldwide [1]. Despite significant advances in cancer treatment, improvements in overall survival in OC have only been modest. [2,3]. Consistent with other malignant tumours, early detection has decreased treatment-associated morbidity and improved survival. More advanced malignancies, particularly those with nodal metastases, have much poorer prognoses, and require more invasive therapy. There is no universal, easy to use screening method for OC, in contrast to colorectal cancer (faecal occult blood) [4], or prostate cancer (prostate specific antigen) [5]. 

The widely accepted risk factors for carcinogenesis of OC are tobacco consumption use and alcohol consumption. Ample evidence suggests that the oral potentially malignant disorders (OPMD), which include leukoplakia, erythroplakia, oral lichen planus, oral submucous fibrosis, actinic cheilosis, and snuff patch, may also contribute to the development of OC [6]. In addition, human papillomavirus (HPV), an etiologic agent for cervical and oropharyngeal carcinoma (OPC) (which includes the tonsillar area, the base of tongue, the soft palate and the oropharynx), is a potential risk factor for OC. Over 200 types of HPV have been reported; however, only high-risk HPV types are associated with cancer development and progression. HPV16 is the most common high-risk HPV type, accounting for the majority of cases of cervical cancer and OPC [7,8]. 

Progression of precursor lesions of HPV associated to invasive carcinomas is closely related with integration of HPV DNA into the human genome [9]. Most clearly, HPV integration events are frequently detected in high-grade cervical intraepithelial neoplasia (CIN) [10]. HPV integration usually results in the deregulation of the E2 opening reading frame (ORF), leading to reduced E2 expression and upregulation of expression of the viral E6 and E7 oncogenes which can abrogate the cell cycle checkpoint and induce genomic instability [11]. However, any role of HPV integration in OC remains to be elucidated. It is well established that p16INK4a (cyclin-dependent kinase 2a) immunohistochemical (IHC) staining can only be a surrogate biomarker of HPV for OPC patients, not for other HNC patients [12]. Accumulating evidence suggests that the importance of additional HPV testing in HPV-associated cancer diagnosis and prognosis [13]. Previous studies have demonstrated that the presence of HPV DNA in tumour tissues is significantly correlated with HPV DNA positivity in saliva samples collected from OPC patients, thus suggesting the use of salivary HPV as a biomarker for the detection of HPV DNA in OPC patients [14,15].

While HPV plays a significant role in OPC, to date it is unclear to what extent this also applies to OC. The first aim of this study was to determine the HPV16 prevalence in OPMD and OC using different sampling methods (unstimulated saliva, oral rinse, oral swab and tumour biopsies). The second aim was to investigate any associations between oral HPV16 infection and lifestyle risk factors (i.e., smoking and drinking) in OPMD and OC. The final aim was to investigate whether the integration of HPV DNA into the human genome was a critical factor in oral carcinogenesis.

## 2. Materials and Methods

### 2.1. Study Design

**Figure 1:**
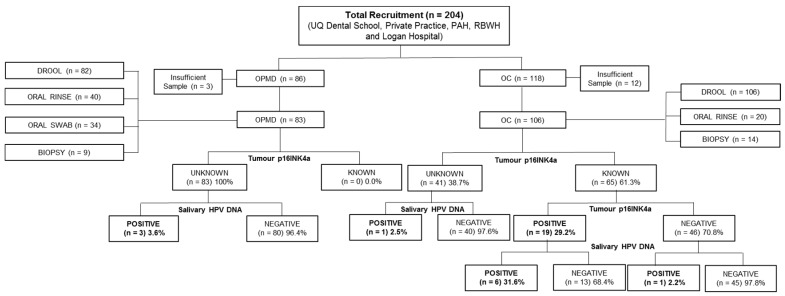
Flow chart of study recruitment.

This study was approved by institutional ethics committees from the University of Queensland [HREC No: 2014000679 and 2014000862], Queensland University of Technology [HREC No: 1400000617 and 1400000641], the Princess Alexandra Hospital [HREC Number: HREC/12/QPAH/381] and the Royal Brisbane and Women’s Hospital [HREC/16/QRBW/447]. A total of 204 patients who had been diagnosed with OPMD (*n* = 86) or OC (*n* = 118) were recruited from the Royal Brisbane and Women’s Hospital, Logan Hospital, Princess Alexandra Hospital, University of Queensland Dental School clinics, and private dental practices (Figure 1). The p16INK4a status in patients with OC lesion was evaluated by Queensland pathologists using CINtec^®^ p16INK4a Histology Kit (E6H4 clone) (Roche MTM Laboratories, Heidelberg, Germany). p16INK4a was considered positive when there was a strong, diffuse nuclear and cytoplasmic staining pattern in the majority (>70%) of tumour cells. The clinical stages of OC patients were classified according to the American Joint Committee on Cancer (AJCC, version 8) based on Tumour–Nodal–Metastasis (TNM). All participants gave written informed consent prior to sampling.

### 2.2. Collection of Unstimulated Saliva, Oral Rinse, Oral Swab and Tissue Samples

The number and type of samples that were obtained are depicted in Figure 1. Unstimulated saliva samples were collected from patients with OPMD (*n* = 85) and OC (*n* = 118), as described in our previous studies [16,17]. Briefly, participants were asked to pool saliva in the mouth (2–5 min) before drooling into a Falcon tube. 

Oral rinse samples were collected from patients with OPMD (*n* = 40) and OC (*n* = 20), using methods employed previously. Briefly, participants were asked to swish and gargle for 1–2 min with 2 × 10 mL 0.9% saline.

Oral swab samples were collected from patients with OPMD (*n* = 34). Participants were asked to rake along the inside of their buccal mucosa with a DNA.SAL collection device (Oasis Diagnostics^®^, Vancouver, WA, USA) for 30–60 s.

Tumour biopsies were obtained from patients with OPMD (*n* = 9) and OC (*n* = 14) after surgical resection. All samples were frozen immediately upon collection and transported back to the laboratory for subsequent processing. 

### 2.3. DNA Isolation

Total DNA was extracted from the unstimulated saliva, oral rinse samples, oral swab samples and tumour biopsies using the QIAmp DNA Mini Kit (Qiagen, Germantown, MD, USA) as per the manufacturer’s protocol. Briefly, 200 μL volumes of lysis buffer was added to the cell pellets with proteinase K and incubated at 56 °C for 10 min. For tumour biopsies, 200 μL of tissue lysis buffer was added with Proteinase K and incubated at 56 °C until the pellets were completely lysed. Then, 200 μL lysis buffer was added to the mixture and incubated at 70 °C for 10 min. After lysis, 200 μL volumes of 100% ethanol was added to the mixture, which was then transferred to QIAmp DNA mini spin columns, following the manufacturer’s instructions. 

### 2.4. Low and High-Risk HPV DNA Genotyping 

To investigate the HPV type distribution in patients with OPMD and OC, a high-throughput MALDI-TOF mass spectrometry-based method (Mass Array Platform, Agena Bioscience, Hamburg, Germany) was used, as previously described [18]. This method is able to detect 16 HPV DNA types (HPV16, 18, 31, 33, 35, 39, 45, 51, 52, 53, 56, 58, 59, 66, 68 and 73) in a single well.

### 2.5. HPV16 DNA Nested PCR

For a sensitive and specific HPV16 PCR, two pairs of primers that flank the region of HPV16 E6 opening reading frame (ORF) were designed. Diluted DNA samples (70 ng) were added to the first PCR master mix containing 10× PCR buffer, 50 mM MgCl2, 10 mM dNTP (No dTTP), 10 mM dUTP, 10 µM HPV16 1F (GTTTCAGGACCCACAGGAGC), 10 µM HPV16 1R (GTCATATACCTCACGTCGCAGT), Uracil DNA Glycosylase (UDG) and Platinum Taq DNA polymerase (Thermo Fisher Scientific, Waltham, MA, USA) in a total volume of 10 μL. The first PCR used the following conditions: 50 °C for 10 min, 95 °C for 10 min; 25 cycles at 95 °C for 30 sec, 60 °C for 30 sec and 72 °C for 30 sec. Next, 2 μL of the first PCR product was used as the template for the second PCR, with 10 µM HPV16 2F (CAGGAGCGACCCAGAAAGTT) and 10 µM HPV16 2R (ACTGTTGCTTGCAGTACACAC), and in the absence of UDG. The second PCR conditions were as follows: 95 °C for 2 min; 30 cycles at 95 °C for 30 sec, 65 °C for 30 sec and 72 °C for 30 sec; and a final extension at 72 °C for 5 min. Human β-globin (Forward: CAACTTCCACGGTTCACC; Reverse: GAAGAGCCAAGGACAGGTAC) was used as an internal control. The second PCR products were subjected to gel electrophoresis. Gel images were recorded using the ChemiDoc™ Gel Imaging System (Bio-rad, Hercules, CA, USA). HPV16 positivity was further confirmed by using Sanger sequencing of the PCR products.

### 2.6. HPV16 E2 and E6/7 DNA qPCR Analysis

Diluted DNA samples (50 ng) were used in duplicate in qPCR. The qPCR assay was carried out with the QuantStudio™ 7 Flex Real-Time PCR System (Applied Biosystems, Foster City, CA, USA). Forward and reverse primers targeted against the region of HPV16 E2 (Forward: AACGAAGTATCCTCTCCTGAAATTATTAG; Reverse: CCAAGGCGACGGCTTTG) and E6/7 (Forward: ACCGGTCGATGTATGTCTTGTTG; Reverse: GATCAGTTGTCTCTGGTTGCAAATC) OFR were used. Human β-globin was used as an internal control. HPV16 E2 and E6 DNA standard calibration curves were generated by using qPCR, respectively, by plotting threshold cycle (Ct values) against the logarithm of the copy number of eightfold serially diluted (1 × 108 to 1 × 101 copies) of pHPV-16 plasmid DNA (American Type Culture Collection (ATCC)^®^ 45113™). Each of the eight-point dilutions were given the same amount of total DNA by spiking with HPV16 negative HNC cell line DNA (SCC-9). Samples were classified as HPV positive when a Ct value for either E2 or E6 was obtained, and as HPV negative when both E2 and E6 Ct values were undetermined: these were assigned a value of 40. 

### 2.7. Physical Status of HPV16

The HPV16 physical status in HPV16 positive OPMD and OC patients (*n* = 11) was classified based on the ratio of E2 and E6/7 copy numbers, as described previously [19,20,21]. HPV16 was regarded as a purely episomal form when the ratio of E2 and E6/7 was equal to or more than 1, in a mixed form with an increased E6/7 copy number (ratio < 1), and in a purely integrated form when E2 was absent.

### 2.8. Statistical Analysis

Fisher’s exact test was used to measure the significance of differences in age, race, sex, drinking and smoking status and oral HPV16 infection between OPMD and OC patients, and between HPV16 negative and positive cases. The odds ratio (OR) with 95% confidence interval (CI) was calculated and used to estimate the relationship between HPV16 status and OC. The non-parametric Mann–Whitney U test was used to compare the differences in HPV16 viral load between OPMD and OC. The Cohen’s kappa coefficient with 95% CI was used to measure the inter-rater agreement between salivary HPV16 DNA testing and p16INK4a expression (https://www.graphpad.com/quickcalcs/kappa1.cfm). The sensitivity, specificity, positive predictive values (PPV), negative predictive values (NPV) and their 95% confidence intervals were calculated. All statistical tests were two-sided, and p-values less than 0.05 were considered significant. Statistical analyses were performed using GraphPad Prism 7 software version 7 (GraphPad Software Inc., La Jolla, CA, USA).

## 3. Result

### 3.1. Patient Characteristics

The details of patients with OPMD (*n* = 83) and OC (*n* = 106) who could provide a sufficient amount of DNA are summarized in Table 1, Supplementary Tables S1 and S2. Most OPMD and OC patients were of Caucasian ethnicity and aged 55 years or above. OC cases were more prevalent in males (*p* = 0.01), ever-smokers (current and former smokers) (*p* = 0.03) and ever-drinkers (standard drink > 7 per week) (*p* = 0.003) when compared to OPMD cases. Detection of HPV16 was rare in both OC and OPMD, and while it was more common in OC than OPMD [*n* = 8 (7.5%) versus *n* = 3 (3.6%), OR 2.18, 95% CI 0.56–8.48, *p* = 0.26], this did not reach statistical significance. HPV16 in saliva fractions (unstimulated saliva/oral rinse) correlated positively with the detection of HPV DNA in tumour biopsies of patients with OPMD and OC (*n* = 23) (Supplementary Tables S3 and S4).

### 3.2. Characteristics of HPV16-Positive Patients with Oral Lesions

Patient clinical and epidemiological characteristics in relation to oral HPV16 infection are detailed in Table 2. There were no significant differences in age, sex and drinking status between the HPV16-negative and -positive groups. All 11 HPV16-positive patients with oral lesions (OC and OPMD) were ever-smokers, compared with 70% (124/177) of HPV16-negative patients (*p* = 0.036).

### 3.3. HPV16 DNA Physical Status and Viral Loads in OPMD and OC

**Figure 2:**
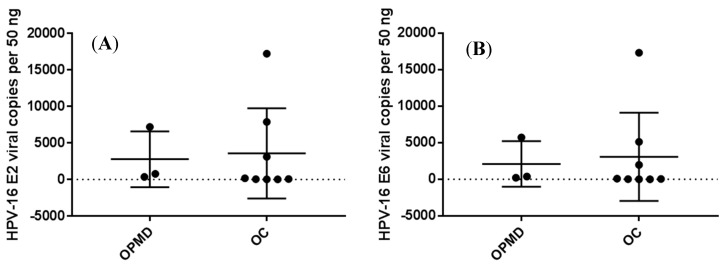
Salivary HPV16 E2 (**A**) and E6 (**B**) viral copies in OPMD and OC patients.

A purely episomal form of HPV16 with the E2/E6 ratio equal to or more than 1 was detected in all 11 HPV16-positive OPMD and OC cases, as summarized in Table 3. HPV16-positive OPMD and OC cases had similar HPV16 E2 and E6/7 viral loads, as shown in Figure 2.

### 3.4. Tumour p16INK4a Expression and Oral HPV16 Infection in OC

65 (61%) OC biopsies were examined by p16INK4a IHC analysis. A total of 19 out of 65 (29%) OC biopsies were positive for p16INK4a staining, while 46 (71%) were negative. Among the 19 p16INK4a-positive OC cases, 6 (32%) were positive for HPV16 DNA. On the other hand, 1 of the 46 (2%) p16INK4a-negative OC cases was positive for HPV16 DNA. The inter-rater agreement between tumour p16INK4a expression and oral HPV16 infection was considered as fair (k = 0.361 95% CI [0.121;0.601]) for OC, as shown in Table 4. 

Sensitivity 0.32 (0.15,0.54), Specificity 0.98 (0.89,1.00), Positive Predictive Value (PPV) 0.86 (0.49,1.00), Negative Predictive Value (NPV) 0.78 (0.65, 0.86).

## 4. Discussion

HPV is the most common sexually transmitted infection, causing an estimated 630,000 (4.5%) new cancer cases globally [1,22]. Growing evidence supports the concept that oncogenic type HPV16 is responsible for most cases of cervical cancer and OPC [23,24]. The role of HPV in the development of cancers in other regions of the mouth is still unclear. To our knowledge, this study is the largest Australian population-based cohort investigation into the possible relationship between oral HPV infection and the risk of OC. Our results show non-significantly higher HPV16 prevalence in OC than in OPMD. In addition, HPV16 positivity was significantly associated with heavy tobacco consumption. Intriguingly, only the purely episomal HPV16 form was detected in all HPV16 positive OPMD and OC cases. No appreciable difference in viral load was found between HPV16 positive OPMD and OC cases. Together, these data suggest that HPV is unlikely to play an etiological role in oral carcinogenesis. 

The reported HPV prevalence in both OPMD and OC varies from 0% to 100% [25,26]. This extreme variation may be explained by several factors, including different ethnicity and variations in sampling methods, as well as in the techniques used for the detection of HPV [27,28]. For Australia, there is no published literature on HPV prevalence rates in OPMD. We found a relatively low prevalence of HPV in OPMD patients when compared to the majority of previous studies [29,30]. However, similar findings have been shown in a recent Hungarian study, which reported a low level of HPV DNA positivity in saliva samples from OPMD patients. Nevertheless, our reported HPV prevalence rate in OC patients is concordant with the findings of previous studies from other western countries [31,32,33] including a recent Australian study by Emmett et al. [34]. More importantly, our results also corroborate the findings of previous investigations in other countries, including Hungary [35], Iran [36], Kazakhstan [37], Romania [38], South Korea [39] and Thailand [40]. However, there was no statistically significant difference in HPV prevalence between OPMD and OC in this Australian cohort.

We found no statistically significant association between oral HPV16 infection and socio-demographic parameters, except for tobacco consumption. Heavy tobacco consumption has been found to facilitate HPV viral persistence, which may contribute to the malignant transformation of cervical cancer [41,42]. However, the current literature on the association between heavy tobacco consumption and HPV-positive HNC is not well delineated [43,44,45]. Several studies suggest that there is an additive or synergistic interaction between heavy tobacco consumption and an elevated risk of developing persistent oral HPV infection and HPV-associated HNC [46,47]. Similar findings are observed in our current study, showing that heavy tobacco consumption is strongly associated with HPV16-positive patient with OPMD and OC lesions.

High-risk HPV DNA integration into the human cellular genome, followed by the upregulation of E6 and E7, often trigger malignant transformation of cells in cervical and anal cancer as well as in OPC [48,49]. Our previous work indicated that most HPV16 OPC cases showed evidence of integration (either partially or fully) in both saliva and tumour fractions [15,50]. However, little attention to date has been paid to investigate whether HPV integration is a necessary step in the carcinogenesis of OC. Our real-time PCR results showed no integration events in both OPMD and OC, according to the HPV16 E2/E6 ratio. Similar findings of low HPV integration rates have been reported in OC patients when compared to OPC patients [49,51]. This is further supported by previous reports of no statistically significant differences in prognostic and survival outcomes between HPV-negative and -positive patients with non-OPC conditions, including OC [52,53,54]. Indeed, a recent study demonstrated that HPV is uncommonly associated with the oncogenesis of OC [55]. Furthermore, both HPV16-positive OPMD and OC had similar viral loads. Thus, the potential etiologic role of HPV in oral carcinogenesis seems rather modest. 

Based on decades of accumulated evidence, accurate HPV testing is crucial for clinical decision making and treatment planning in HNC patients [56]. The current ‘gold standard’ of p16INK4a staining that is used for the detection of HPV in patients with non-OPC and OPMD has been shown to have several drawbacks [57,58,59]. A large multicentre study reported a discordance between p16INK4a expression and HPV infection in patients with non-oropharyngeal HNC (n = 683), including subsites of the oral cavity, hypopharynx, and larynx [58]. This is further supported by a recent study by Zafereo et al. [60] which indicated that only six of the 40 p16INK4a-positive OC cases had detectable HPV DNA, whilst 31 of the 110 p16INK4a-negative OC cases were HPV DNA positive. This suggests that the overexpression of p16INK4a is not a reliable surrogate marker of HPV in OC. Similar findings of low concordance were made in the current study. The underlying mechanism of p16INK4a overexpression in OC may not be driven solely by HPV infection. If this is indeed the case, using p16INK4a as a standalone marker for the detection of HPV in OC would not be recommended. 

The major limitations of the present study are the relatively small sample sizes, the limited availability of fresh tumour samples of OPMD and OC cases, and the use of saliva for HPV testing which might not reflect the HPV positivity in both OPMD and OC samples. Furthermore, additional studies will be needed to confirm whether our findings apply to all regions in Australia. In this study, p16INK4a IHC analysis was only performed in OC tumour samples with features suspicious of HPV infection. Finally, an additional robust method is needed for future studies of HPV integration events. 

## 5. Conclusions

In conclusion, we found a low oral HPV16 prevalence in both OPMD and OC. Interestingly, we observed a statistically significant association between oral HPV16 infection and heavy tobacco consumption. However, no integration events occurred in both OPMD and OC cases that were examined. Taken together, our results do not support a major role of HPV in oral carcinogenesis. 

## Figures and Tables

**Table 1:** Patient Demographics.

Variables	Categories	OC (N = 106)		OPMD (N = 83)		*p*-Value
		No.	%	No.	%	
**Age (years)**	≤55	17	16.0	20	24.1	0.2
	>55	89	84.0	63	75.9	
**Sex**	Male	75	70.8	43	51.8	0.01
	Female	31	29.2	40	48.2	
**Smoking Status**	Ever	83	78.3	52	62.7	0.03
	Never	23	21.7	30	36.1	
	Unknown	0	0.0	1	1.2	
**Drinking Status**	Ever	49	46.2	19	22.9	0.003
	Never	55	51.9	57	68.7	
	Unknown	2	1.9	7	8.4	
**HPV16 Infection**	Yes	8	7.5	3	3.6	0.35
	No	98	92.5	80	96.4	

**Table 2:** Differences between HPV16 negative and HPV16 positive patients with oral lesions.

Variables	Categories	HPV16 Negative		HPV16 Positive		*p*-Value
		No.	%	No.	%	
**Age (years)**	≤55	35	19.7	2	18.2	>1.000
	>55	143	80.3	9	81.8	
**Sex**	Male	109	61.2	9	81.8	0.214
	Female	69	38.8	2	18.2	
**Smoking Status**	Ever	124	70.1	11	100.0	0.036
	Never	53	29.9	0	0.0	
**Drinking Status**	Ever	62	36.7	6	54.5	0.336
	Never	107	63.3	5	45.5	

**Table 3:** Salivary HPV16 viral load and viral physical status in oral potentially malignant disorder (OPMD) and oral cavity carcinoma (OC) patients.

OC	Genotype	E2 CT	E2 Viral Copies per 50 ng	E6 CT	E6 Viral Copies per 50 ng	E2/E6 Ratio	Physical Status
OC 2	16	22.07	7882.79	21.52	5114.37	1.54	Episomal
OC 46	16	27.71	180.35	27.39	103.83	1.74	Episomal
OC 49	16	20.90	17,245.15	19.69	17,296.29	1.00	Episomal
OC 51	16	30.92	21.03	31.34	7.56	2.78	Episomal
OC 54	16	30.90	21.24	30.21	15.97	1.33	Episomal
OC 70	16	29.40	58.02	28.30	56.77	1.02	Episomal
OC 84	16	23.45	3124.61	22.97	1963.43	1.59	Episomal
OC 94	16	28.95	78.37	29.51	25.40	3.09	Episomal
OPMD 6	16	22.21	7188.51	21.36	5709.28	1.26	Episomal
OPMD 11	16	25.50	790.81	25.40	390.00	2.03	Episomal
OPMD 30	16	26.68	358.78	26.31	213.20	1.68	Episomal

**Table 4:** Oral HPV16 infection and tumour p16INK4a in OC patients.

Salivary HPV16 DNA Status	p16INK4a Status	
	Positive	Negative
Positive	6 (31.6%)	1 (2.2%)
Negative	13 (68.4%)	45 (97.8%)

## Supplementary Materials

The following are available online at https://www.mdpi.com/2218-273X/10/2/223/s1. Table S1: Clinical characteristics of OPMD. Table S2: Clinical characteristics of OC. Table S3: HPV-16 genotyping in unstimulated saliva, oral rinse, oral swab and tumour biopsies of OPMD patients. Table S4: HPV-16 infection (saliva and tumour biopsy) and tumour p16INK4a in OC patients.
