# Supplementary material for: Oral HPV16 Prevalence in Oral Potentially Malignant Disorders and Oral Cavity Cancers
Source: Biomolecules. 2020 Feb 3;10(2):223. doi: 10.3390/biom10020223 (PMC7072384; doi:10.3390/biom10020223)
Supplement: Supplementary file 1 [file biomolecules-10-00223-s001.pdf]

**Supplementary Table S1: Clinical characteristics of OPMD.**

| | **OPMD (n = 83)** |
|---|---|
| **Diagnosis** | |
| Benign squamous papilloma | 3 (3.6) |
| Oral Leukoplakia | 22 (26.5) |
| Oral Lichen Planus | 9 (10.8) |
| Oral Lichenoid Lesion | 5 (6.0) |
| Dysplasia | 40 (48.2) |
| NA | 4 (4.8) |
| | |
| **Anatomic sites** | |
| Tongue | 33 (39.8) |
| Floor of mouth | 6 (7.2) |
| Alveolus | 6 (7.2) |
| Buccal mucosa | 16 (19.3) |
| Retromolar trigone | 1 (1.2) |
| Hard palate | 3 (3.6) |
| Others | 4 (4.8) |
| N/A | 14 (16.9) |

**Supplementary Table S2: Clinical characteristics of OC.**

| | **OC (n = 106)** |
|---|---|
| **AJCC TNM** | |
| Stage I | 21 (19.8) |
| Stage II | 27 (25.5) |
| Stage III | 7 (6.6) |
| Stage IV | 35 (33.0) |
| NA | 16 (15.1) |
| | |
| **Anatomic sites** | |
| Tongue | 51 (48.1) |
| Floor of mouth | 22 (20.8) |
| Alveolus | 15 (14.2) |
| Buccal mucosa | 6 (5.7) |
| Retromolar trigone | 7 (6.6) |
| Hard palate | 5 (4.7) |

**Supplementary Table S3: HPV-16 genotyping in unstimulated saliva, oral rinse, oral swab and tumour biopsies of OPMD patients.**

| Sample | Unstimulated saliva | Oral rinse | Oral swab | Tumour biopsy |
|---|---|---|---|---|
| OPMD 12 | No | No | No | No |
| OPMD 14 | No | No | No | No |
| OPMD 18 | No | No | No | No |
| OPMD 21 | No | No | Insufficient DNA | No |
| OPMD 24 | No | No | Insufficient DNA | No |
| OPMD 25 | No | No | No | No |
| OPMD 29 | No | No | No | No |
| OPMD 30 | Yes | Yes | No | Yes |
| OPMD 31 | No | No | No | No |

**Supplementary Table S4: HPV-16 infection (saliva and tumour biopsy) and tumour p16INK4a in OC patients.**

| Sample | Saliva | Tumour biopsy | p16INK4a status |
|---|---|---|---|
| OC 1 | No | No | Negative |
| OC 2 | Yes | Yes | Positive |
| OC 3 | No | No | Negative |
| OC 4 | No | No | Negative |
| OC 7 | No | No | Positive |
| OC 8 | No | No | Negative |
| OC 9 | No | No | Negative |
| OC 11 | No | No | Negative |
| OC 69 | No | No | UKNOWN |
| OC 72 | No | No | Positive |
| OC 76 | No | No | UKNOWN |
| OC 79 | No | No | UKNOWN |
| OC 85 | No | No | UKNOWN |
| OC 87 | No | No | Positive |
